# Intestinal fatty acid-binding protein (I-FABP) at ICU admission as early biomarker of intestinal injury and predictor of mortality in septic shock

**DOI:** 10.1186/s40635-026-00963-9

**Published:** 2026-08-26

**Authors:** Thorben Pape, Pedro David Wendel-Garcia, Nina Rittgerodt, Bahar Nalbant, Jan Hinrichs, Lena S. Becker, Anna M. Hunkemöller, Jakob Raith, Sören K. J. Boblitz, Jannik Ruwisch, Pia-Victoria Fangmann, Heiner Wedemeyer, Marius M. Hoeper, Christian Bode, Sascha David, Klaus Stahl, Benjamin Seeliger

**Affiliations:** 1 Department of Respiratory Medicine and Infectious Diseases, Hannover Medical School, Carl-Neuberg-Str.1, 30625 Hannover, Germany; 2 Biomedical Research in Endstage and Obstructive Lung Disease Hannover (BREATH), German Center for Lung Research (DZL), Hannover, Germany; 3 PRACTIS Clinician Scientist Program, Dean’s Office for Academic Career Development, Hannover Medical School, Hannover, Germany; 4 Institute for Intensive Care Medicine, University Hospital Zurich and University of Zurich, Zurich, Switzerland; 5 Division of Cardiothoracic Anesthesia and Intensive Care Medicine, Department of Anesthesiology, General Intensive Care and Pain Medicine, Medical University of Vienna and Vienna General Hospital, Vienna, Austria; 6 Department of Gastroenterology, Hepatology, Infectious Diseases and Endocrinology, Hannover Medical School, Hannover, Germany; 7 Institute of Diagnostic and Interventional Radiology, Hannover Medical School, Hannover, Germany; 8 Department for Diagnostic and Interventional Radiology and Neuroradiology, St. Bernward Hospital Hildesheim, Hildesheim, Germany; 9 Department of Radiology and Biomedical Imaging, Section of Interventional Radiology, Yale School of Medicine, 330 Cedar St, TE-2, New Haven, CT 06520-8055 USA; 10 Department of Anesthesiology and Intensive Care Medicine, University Hospital Bonn, Bonn, Germany; 11 Department of Nephrology and Hypertension, Hannover Medical School, Hannover, Germany

**Keywords:** Ischemic bowl disease, Gut, Gut injury, NOMI, Sepsis

## Abstract

**Background:**

Septic shock and acute respiratory distress syndrome (ARDS) are major causes of mortality. Intestinal hypoperfusion is a frequent but often unrecognized complication, and the timing and extent of intestinal injury in these syndromes remain unclear. Intestinal fatty acid-binding protein (I-FABP) has emerged as a potential biomarker of intestinal injury. This study aimed to define a clinically relevant I-FABP cutoff for predicting relevant intestinal injury and to assess its association with disease severity and outcome.

**Methods:**

We performed a post-hoc analysis combining cohorts of critically ill patients from the prospective EXCHANGE-pilot, the RCT EXCHANGE-1, and the SPARE-14 registry. The analysis included 102 patients with septic shock and/or ARDS, 22 patients with non-occlusive mesenteric ischemia (NOMI) from the REPERFUSE study, and 20 healthy individuals. Circulating I-FABP concentrations were measured by ELISA in plasma samples obtained at ICU admission. Receiver operating characteristic (ROC) analysis was used to define the optimal I-FABP threshold for predicting intestinal hypoperfusion, using patients with NOMI as positive- and healthy individuals as negative controls. Associations between I-FABP, disease severity, and 28-day mortality were analyzed.

**Results:**

ROC analysis identified an initial I-FABP cutoff of 1070 pg/ml (sensitivity 68%, specificity 100%) for the development of intestinal ischemia defined by criteria of manifest NOMI. I-FABP levels at ICU admission were significantly elevated in patients with ARDS and/or septic shock compared with healthy controls (1108 pg/ml (IQR 291–2846) vs. 479 pg/ml (IQR 358–631), *p* = 0.03), and comparable to those in NOMI patients. Exploratory generalized additive model analyses indicated a non-linear association between I-FABP concentrations and established markers of shock severity. Patients with high I-FABP levels (above 1070 pg/ml) had a higher 28-day mortality (high: 52.9% vs. low: 33.3%, *p* = 0.02). In a multivariate logistic regression model, I-FABP and SOFA score remained independently associated with 28-day mortality (I-FABP: adjusted OR 1.27 (1.03–1.62), *p* = 0.023), without independent association of lactate concentration.

**Conclusion:**

Elevated plasma I-FABP may identify undetected intestinal injury in critically ill patients with septic shock and/or ARDS and might be associated with higher 28-day mortality. Additionally, I-FABP might reflect a distinct facet of critical illness that is not fully captured by conventional measures of organ dysfunction or shock severity and I-FABP concentrations ≥ 1070 pg/ml may help identify patients at high risk for intestinal ischemia who could benefit from early therapeutic interventions.

**Supplementary Information:**

The online version contains supplementary material available at 10.1186/s40635-026-00963-9.

## Background

Sepsis, septic shock, and acute respiratory distress syndrome (ARDS) remain leading causes of morbidity and mortality in the intensive care unit. According to the Sepsis-3 definition, sepsis is a life-threatening organ dysfunction resulting from a dysregulated host response to an infection [1]. Sepsis is the most common precipitating cause of ARDS, and both syndromes share overlapping inflammatory and endothelial injury pathways that contribute to both multiorgan dysfunction and death [2–5]. The gastrointestinal tract has long been described as a “motor” of multiple organ failure, but the extent and timing of gut injury and bacterial translocation remain incompletely understood [6]. An increasing number of acute mesenteric ischemia is reported in ARDS patients, especially Covid-19 patients [7, 8]. Clinical signs of intestinal ischemia or barrier dysfunction are often subtle or absent in sedated or vasopressor-dependent patients, and diagnostic imaging has limited sensitivity for early mucosal injury [7, 9]. Non-occlusive mesenteric ischemia (NOMI) represents the most severe form of intestinal hypoperfusion, characterized by mesenteric vasospasms without major vascular obstruction, leading to intestinal ischemia associated with an exceedingly high mortality of up to 90% [10–12]. Early diagnosis of NOMI is crucial because selected patients may benefit from targeted intra-arterial vasodilatory therapy [13–15].

Biomarkers of enterocyte damage, such as intestinal fatty acid-binding protein (I-FABP) (mucosal intestinal damage), have been proposed as surrogate indicators of intestinal injury in critically ill patients. Yet, their diagnostic accuracy and clinical relevance across heterogeneous ICU populations remain uncertain and they have not been implemented in clinical routine [16–23]. The identification of an early, reliable marker of intestinal injury could enable timely gut-directed interventions before irreversible mesenteric ischemia develops.

Intestinal fatty acid‑binding protein (I‑FABP, FABP2) is a middle (≈ 14–15 kDa) cytosolic protein highly expressed in mature enterocytes of the small intestinal mucosa [24, 25]. It facilitates intracellular binding and trafficking of long‑chain fatty acids, supporting absorption, metabolism, and protection from lipotoxicity [24]. Under physiological conditions, I‑FABP is confined to enterocytes, resulting in very low or undetectable serum levels [25]. When enterocytes are damaged I‑FABP is released, then providing a direct measure of epithelial compromise [26, 27]. Its sensitivity and rapid kinetics make I‑FABP a valuable translational biomarker for detecting (acute) mucosal injury and assessing intestinal barrier integrity [17, 20, 25, 28]. Elevated circulating I-FABP concentrations have been described in patients with NOMI, reflecting acute enterocyte injury [14, 22, 23], however, robust data defining I-FABP thresholds for clinically relevant intestinal injury or predicting response to vasodilatory therapy are scarce [17, 29].

This study aimed to evaluate I-FABP as a biomarker of intestinal injury preceding clinically apparent ischemia in critically ill patients with septic shock and ARDS. Specifically, we sought to (1) define a clinically relevant cutoff value of I-FABP for the detection of significant gut injury; (2) assess the frequency and magnitude of I-FABP elevation across these disease states; and (3) investigate the association of I-FABP levels alone or in combination with clinical parameters as a biomarker of intestinal injury and predictor of mortality.

## Methods

### Study population

Patients with ARDS and/or septic shock were included for this post-hoc analysis from the EXCHANGE-Pilot trial, EXCHANGE-1 RCT and a prospective sepsis and ARDS registry (SPARE-14). Sepsis and septic shock were defined by the Sepsis-3 definition [1]. ARDS was defined by the Berlin definition [4, 5]. A combination of both diseases was seen if criteria for both septic shock and ARDS were fulfilled. For further analysis this cohort was unified as “patients with ARDS and/or septic shock” representing critical ill patients.

### EXCHANGE-Pilot and EXCHANGE-1

Patients from both a prospective single center non-randomized study (EXCHANGE-Pilot trial, recruited from July 2016 to July 2017, clinicaltrials.gov Identifier: NCT03065751) [30] and a prospective, open label RCT (EXCHANGE-1 trial, recruited from June 2018 to July 2020, clinicaltrials.gov, Identifier: NCT04231994) at two university hospitals in Germany [31, 32] were included in this study. Patients with early and severe septic shock (onset < 24 h) requiring a norepinephrine dose of ≥ 0.4 µg/kg/min were included. All patients were treated according to the 2016 Surviving Sepsis Campaign (SSC) guidelines [33]. Patients who were in the therapeutic plasma exchange (TPE) group (68.1% of included patients from EXCHANGE trials) received treatment within six hours of study inclusion. The detailed protocol and main results of the trials have been published elsewhere [30–32].

### Sepsis and ARDS registry (SPARE-14)

Patients with ARDS by the Berlin definition [5] and/or sepsis with and without shock [1] are included into the registry (Sepsis and ARDS Registry (SPARE-14)) from the intensive care unit of Hannover Medical School (MHH) since February 2019 and stored at the MHH’s Unified Biobank. Patients aged above 18 years were included if they were ventilated for less than seven days and fulfilled above criteria. Patients were excluded if their ICU stay surpassed 96 h at time of screening, if the diagnosis of sepsis had been established for more than 96 h, or in case of pregnancy. For this study patients recruited between January 2019 and December 2023 were included. A total of 17 (31%) patients received TPE as an individual treatment option.

### REPERFUSE

In order to obtain patients with severe ischemic gut injury as positive controls, we used blood samples and clinical data from the REPERFUSE study. The REPERFUSE study was a prospective, observational, monocentric study investigating outcomes following local intra-arterial prostaglandin therapy in critically ill patients diagnosed with NOMI (recruited from October 2018 to October 2021, clinicaltrials.gov, Identifier: NCT04235634) [14]. Patients were screened if the following criteria were fulfilled: (1) persistent shock: norepinephrine dose > 0.2 µg/kg/min over > 48 h AND (2) intestinal failure: paralytic ileus > 24 h despite prokinetic therapy AND (3) new onset of progressive organ failure (≥ 2 out of 6 predefined criteria). Patients were then included into the study when a definitive diagnosis of NOMI was made by characteristic imaging findings by either CT-angiography or/and mesenteric digital subtraction angiography (DSA). The detailed protocol and main results of the trials have been published elsewhere [14]. Of note, we have recruited controls who reported to be healthy on a voluntary basis in 2021 during the REPERFUSE study. 55% were female with a median age of 29 (25–32) years. Blood was drawn at one timepoint.

### Ethical approval

The study was performed according to the ethical standards that were laid down in the Declaration of Helsinki 1964 and its later amendments. Informed consent was obtained from patients and/or legal representatives accordingly. The study was approved by the according institutional review boards (Hannover Medical School: No. 2786 − 2015 (EXCHANGE-Pilot) and No. 8852_MPG_23b_2020 (EXCHANGE-1), No. 8092_BO_S_2018 (REPERFUSE), No. 8146_BO_K_2018, No. 9720_BO_K_2021 (SPARE-14 registry); University Hospital Bonn (No. 024/20) (EXCHANGE-1)).

### Blood sample collection and measurement of parameters

Blood was taken at study inclusion (EXCHANGE-Pilot, EXCHANGE-1, SPARE-14 registry: *n* = 102 patients with ARDS and/or septic shock, REPERFUSE: *n* = 22 patients with NOMI) as well as from healthy individuals without any acute illness (controls: *n* = 20). The EDTA plasma samples were centrifuged with 3800 rpm for 10 min at 4 °C and stored at − 20 °C. For some patients from SPARE-14 blood was taken on the third day after study inclusion and stored accordingly.

### Data collection

Routine clinical and laboratory data were collected at study inclusion from the electronic medical records including the patient data management system (PDMS). SOFA scores were calculated according to the description by Vincent et al. [34]. Organ failure was defined as an organ specific SOFA score of equal or greater than two. More details concerning data collection are stated in Supplemental Material 1.

Data completeness was assessed for all study variables. Missing data were very limited, particularly for variables included in the primary analyses. Therefore, no imputation procedures were performed, and analyses were conducted using available data.

### I-FABP ELISA

Enzyme-linked immunosorbent assays (ELISA) were performed for intestinal fatty acid binding protein (I-FABP) using the commercially available I-FABP kit (HK406-01, HycultBiotech, Uden, The Netherlands) assay from human plasma. 1:2 dilutions of human plasma were used and the ELISA was performed according to manufacturer’s instructions. Details can be found elsewhere [14].

### Statistical analysis

Categorical variables are shown as numbers (n) and percentages (%). Unless indicated otherwise, continuous variables are shown as median and 25% − 75% quartiles. Variables were checked for normal distribution using the D’Agostino-Pearson omnibus normality test and the Shapiro-Wilk normality test. For comparisons, Mann-Whitney U test, Wilcoxon matched - pairs signed rank test, two-sided paired t-test, one-way ANOVA, Friedmann-test and Kruskal-Wallis with Dunn’s post-test were used accordingly. A Receiver Operating Characteristic (ROC) curve calculation for optimal cutoff of I-FABP predicting gut injury was performed with the former REPERFUSE cohort and healthy controls (R package *pROC*). Patients with significant gut injury were defined by patients with clinical diagnosis of NOMI (angiography findings, endpoint of ROC analysis) from REPERFUSE. To explore potential non-linear associations between I-FABP concentrations and lactate concentrations or norepinephrine dose, generalized additive models (GAMs) with smooth terms were fitted (*mgcv*).

Univariate and multivariate logistic regression (*lme4*) were performed to calculate odds ratios for individual variables. Odds ratios (ORs) with 95% confidence intervals (CIs) were estimated using multivariable logistic regression models. Adjustment variables were selected a priori based on their established clinical relevance as determinants of mortality in critically ill patients. The primary multivariable model included age, SOFA score, renal replacement therapy (RRT), mechanical ventilation and lactate concentration. Given the limited number of mortality events, the number of covariates was intentionally restricted to avoid model overfitting. Model stability (number of mortality events per estimated predictor parameter (EPV)), discrimination (area under the curve), overall predictive accuracy (Brier score) and model fit (Cox–Snell and Nagelkerke R²) were analyzed accordingly. As an additional sensitivity analysis, TPE was included in the multivariable logistic regression model. Survival was visualized by Kaplan-Meier graphs and analyzed by Log-rank and post-hoc tests (R packages *survival* and *survminer*). All reported p-values are two-sided unless indicated otherwise; p-values < 0.05 were considered statistically significant. The R environment for statistical computing (Version 4.1.2, R Foundation for Statistical Computing, Vienna, Austria) was used for data analysis.

## Results

### Optimal threshold for I-FABP indicating ischemic gut injury

**Fig. 1:**
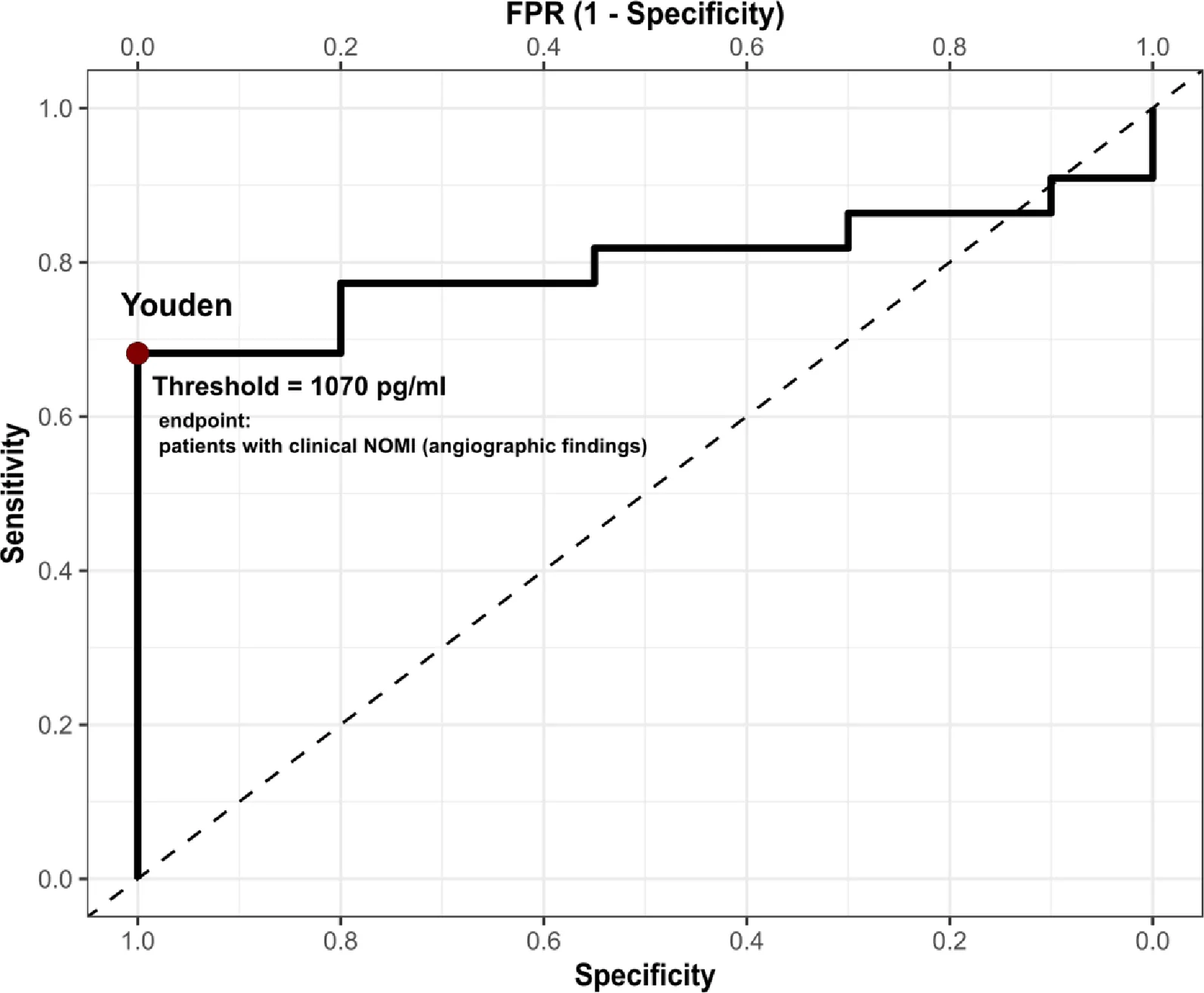
Optimal threshold for I-FABP indicating ischemic gut injury. A Receiver Operating Characteristic (ROC) curve was performed in patients with angiography-confirmed NOMI from the REPERFUSE study and healthy controls (negative controls) in order to find an optimal threshold by Youden indicating intestinal injury

A ROC analysis with previous collected data on patients with NOMI diagnosed by angiography findings and healthy individuals from the REPERFUSE study was performed to find an optimal I-FABP cutoff indicating significant ischemic gut injury. The optimal cutoff by Youden was identified at 1070 pg/ml with a sensitivity of 68% (95% CI: 45–86%) and a specificity of 100% (95% CI: 83–100%) (Fig. 1). The negative predictive value (NPV) was 74% (95% CI: 54–89%), whereas the positive predictive value (PPV) was identified at 100% (95% CI: 78–100%). The area under the curve was 0.80 (0.65–0.95, *p* < 0.001).

### Cohort characterization

To further analyze intestinal injury in critical ill patients with septic shock and/or ARDS, we included patients from our Sepsis and ARDS registry (SPARE-14) and from the previous EXCHANGE-pilot trial and the EXCHANGE-1 RCT.

69% of the patients were male with a median age of 56 (IQR 44–62) years. The causative pathogen was identified in 77% of cases and the most common sides of infection were lungs and abdomen. 91% of patients needed mechanical ventilation with an oxygenation index (PaO_2_ / FiO_2_) of 123 (IQR 89–184) mmHg and renal replacement therapy (RRT) was initiated in 49% of patients. Median norepinephrine dose at inclusion was 0.611 µg/kg/min (IQR 0.368–0.858) and median lactate concentration were 2.7 (IQR 1.7–5.7) mmol/l indicated a high severity of shock. The median SOFA score at study inclusion was 13 (IQR 10–17) points (Table 1).

**Table 1:** Demographic and clinical characteristics at study inclusion

Category (median (IQR) or *N* (%))	All *n* = 124	NOMI *n* = 22	ARDS and/or Septic Shock *n* = 102	*p*
Age (years)	56 (44–62)	55 (43–69)	56 (44–62)	0.556^#^
Sex (female)	43 (34.7)	11 (50.0)	32 (31.4)	0.096^‡^
BMI (kg/m^2^)	25.4 (22.9–29.6)	25.4 (22.0-29.6)	25.4 (22.9–29.4)	0.879^#^
Comorbidities				
Adipositas	34 (32.1)	6 (27.3)	28 (33.3)	0.588^‡^
Hypertension	44 (44.1)	11 (50.0)	33 (38.8)	0.525^‡^
Diabetes	20 (18.7)	3 (13.6)	17 (20.0)	0.588^‡^
COPD	11 (10.3)	1 (4.5)	10 (11.8)	0.453^‡^
Heart insufficiency	19 (17.9)	5 (22.7)	14 (16.7)	0.634^‡^
CAD	18 (16.8)	10 (45.5)	8 (9.4)	**< 0.001** ^‡^
CKD	30 (28.6)	10 (45.5)	20 (24.1)	0.122^‡^
Site of infection				**< 0.001** ^‡^
Pulmonary	75 (60.5)	11 (50.0)	64 (69.6)	0.082^‡^
Abdominal	35 (28.2)	19 (86.4)	16 (17.4)	**< 0.001** ^‡^
Soft tissue	12 (9.7)	6 (27.3)	6 (6.6)	**0.004** ^‡^
Endocarditis	5 (4.0)	3 (13.6)	2 (2.2)	**0.018** ^‡^
Urogenital	3 (2.4)	2 (9.1)	1 (1.1)	**0.035** ^‡^
SOFA score (points)	15 (11–18)	17 (16–18)	13 (10–17)	**0.005** ^#^
Norepinephrine dose (µg/kg/min)	0.561 (0.307–0.804)	0.357 (0.191–0.585)	0.611 (0.368–0.858)	**0.005** ^#^
Lactate concentration (mmol/l)	3.5 (2.0-7.2)	8.8 (5.3–12.2)	2.7 (1.7–5.7)	**< 0.001** ^#^
RRT	68 (55.3)	19 (86.4)	49 (48.5)	**0.001** ^‡^
Mechanical ventilation	109 (88.6)	17 (77.3)	92 (91.1)	0.064^‡^
Oxygenation index (PaO_2_/FiO_2_) (mmHg)	130 (93–208)	211 (140–348)	123 (89–184)	**< 0.001** ^#^
CRP (mg/l)	190 (111–300)	110 (44–156)	220 (141–309)	**< 0.001** ^#^
PCT (µg/l)	13 (3–43)	4 (2–10)	17 (3–52)	**0.037**
WBC (10^3^/µl)	10.6 (6.1–19.1)	10.3 (5.8–19)	11.3 (6.1–19)	0.945^#^

Demographic and clinical characteristics of the whole patient cohort (*n* = 124) at study inclusion and divided by patients with NOMI (*n* = 22) and patients with septic shock and/or ARDS (*n* = 102). Values are presented as median (25% to 75% interquartile range) or if categorical as numbers and percentages.P-Values < 0.05 were seen as significant and displayed in bold. #=Mann-Whitney-U test; ‡=Chi-Squared test

BMI - Body mass index, CAD – coronary artery disease, CKD – chronic kidney disease, COPD – chronic obstructive pulmonary disease, CRP - C-reactive protein, IQR - Interquartile range, PCT - Procalcitonin, RRT - Renal replacement therapy, SOFA - Sequential Organ Failure Assessment, WBC - White blood cell

Comparing this septic shock and/or ARDS cohort to the NOMI cohort [14] used to calculate the I-FABP cutoff, no significant differences concerning demographics at study inclusion were seen. However, coronary artery disease was significantly more often observed in patients with NOMI. Markers of disease severity differed between groups, with higher lactate concentrations but lower norepinephrine doses, CRP and PCT levels, and higher oxygenation indices in NOMI patients. Infection sites also varied, with abdominal, soft tissue, endocardial, and urogenital infections being more frequent in the NOMI group (Table 1). Of note, mortality was 73% in the NOMI cohort and 43% in the pooled ARDS and/or septic shock cohort, as described in more detail below.

### I-FABP was increased in patients with ARDS and/or septic shock

**Fig. 2:**
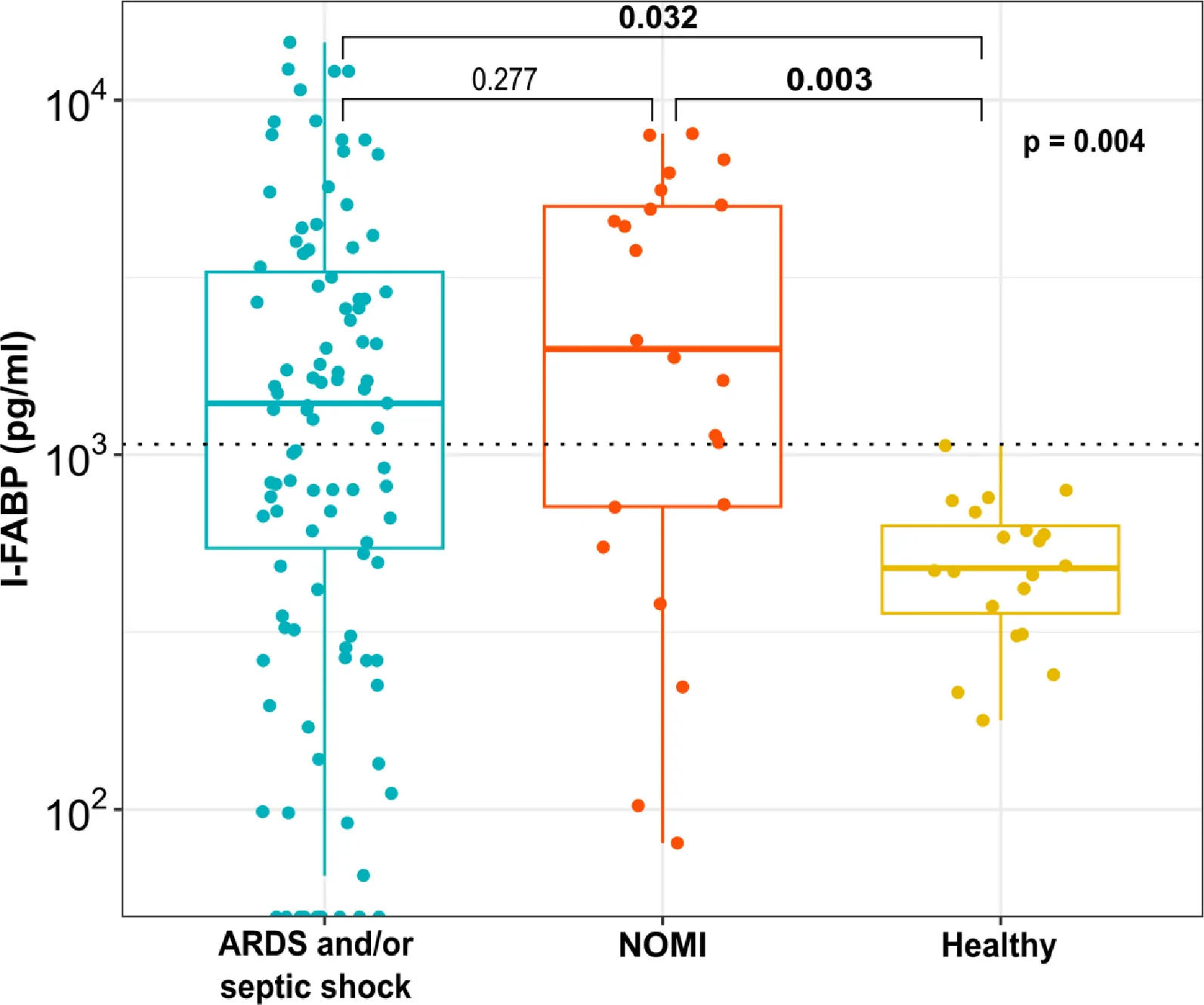
I-FABP was increased in patients with ARDS and/or septic shock. Concentrations of intestinal fatty acid binding protein (I-FABP) are shown for patients with ARDS and/or septic shock, patients with NOMI as well as healthy controls. Optimal threshold indicating intestinal injury is indicated by a dotted line. Values are presented as median (25% to 75% interquartile range). The Kruskal-Wallis test with Dunn’s post hoc test was performed. p-values < 0.05 were considered significant

Median I-FABP was significantly increased in patients with ARDS and/or septic shock compared to healthy controls (ARDS and/or septic shock: 1108 (IQR 291–2846) pg/ml vs. healthy: 479 (IQR 358–631) pg/ml, *p* = 0.032). In NOMI patients (as positive controls) median I-FABP concentrations were also significantly increased compared to healthy individuals (NOMI: 1990 (IQR 715–5023) pg/ml vs. healthy: 479 (IQR 358–631) pg/ml, *p* = 0.003). However, there was no significant difference between ARDS and/or sepsis patients and NOMI patients (*p* = 0.277) (Fig. 2). Dividing ARDS and/or septic shock into subgroups revealed a significant increase of median serum I-FABP levels for patients with septic shock (*p* = 0.012) and patients with ARDS and septic shock (*p* < 0.001) compared to healthy controls. However, patients with ARDS without concomitant septic shock, did not have higher median I-FABP levels compared to healthy individuals (*p* = 0.14) indicating septic shock rather than ARDS as the driver of intestinal injury. (Supplemental Figure S1). When assessing I-FABP trajectories in 27 patients after a median follow-up of 65.75 h ± 14.15 after the baseline samples, no significant change (baseline: 836 (258–2526) vs. follow-up: 1149 (248–2428), *p* = 0.91) was observed overall or within subgroups (Supplemental Figure S2). Of note, TPE was not able to reduce I-FABP significantly during follow-up (*p* = 0.833) (Supplemental Figure S2C).

### Relationship of I-FABP with lactate concentrations and norepinephrine dose as markers of shock severity

**Fig. 3:**
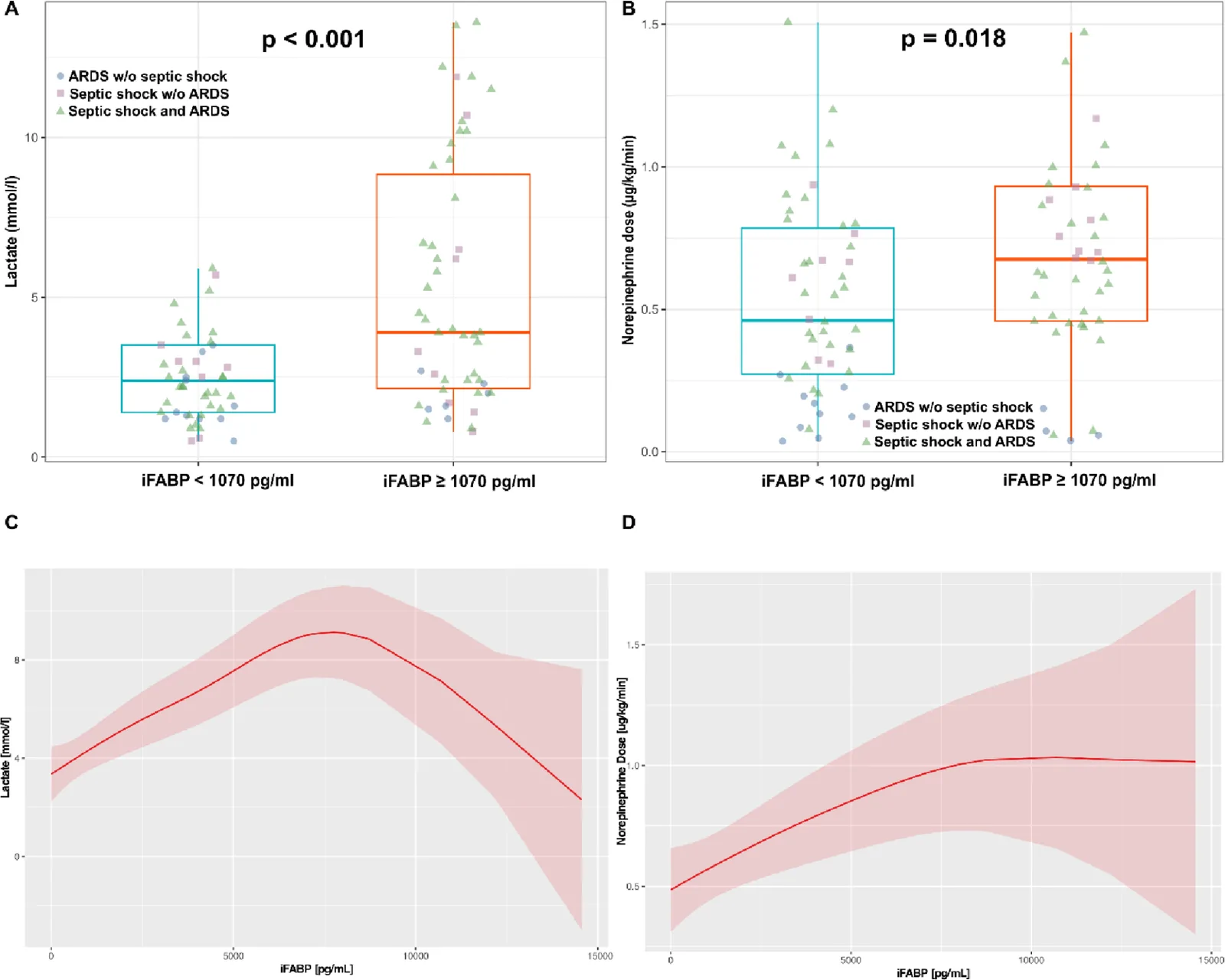
Relationship of I-FABP with lactate concentrations and norepinephrine dose as markers of shock severity. Patients with ARDS and/or septic shock were stratified by low and high I-FABP (threshold 1070 pg/ml) and (**A**) lactate concentrations and (**B**) norepinephrine doses were shown respectively. Mann-Whitney-U test was performed for group comparison. Associations between I-FABP and (**C**) lactate levels or (**D**) norepinephrine dose were estimated using generalized additive models with smooth terms. 95% confidence intervals are shown as shaded areas. p-values < 0.05 were considered significant

Based on the calculated threshold (1070 pg/ml) for significant intestinal injury (low vs. high I-FABP), we correlated circulating I-FABP with lactate concentrations and norepinephrine doses as markers of shock severity in the ARDS and/or septic shock cohort. Both median lactate concentrations (low I-FABP: 2.4 (IQR 1.4–3.5) mmol/l vs. high I-FABP: 3.9 (IQR 2.2–8.9) mmol/l, *p* < 0.001) as well as median norepinephrine doses (low I-FABP: 0.46 (IQR 0.27–0.79) µg/kg/min vs. high I-FABP: 0.68 (IQR 0.46–0.93) µg/kg/min, *p* = 0.018) were significantly increased when I-FABP was found above the identified threshold (Fig. 3A, B).

Estimated smooth functions from generalized additive models suggested non-linear relationships between I-FABP concentrations and lactate concentrations (Fig. 3C) and norepinephrine dose (Fig. 3D). Lactate levels and norepinephrine requirements increased with rising I-FABP concentrations up to approximately 8000 pg/ml, after which both associations appeared to plateau. For lactate, a decline was observed at the highest I-FABP concentrations (Fig. 3C, D).

Patients with high I-FABP concentrations had higher lactate concentrations, higher norepinephrine doses, and higher rates of RRT and TPE. Apart from these differences, demographics, disease severity, comorbidities, and site of infection at study inclusion were comparable between groups. Interestingly, median SOFA score did not differ between the two groups (low I-FABP: 13 (IQR 10–17) vs. high I-FABP: 15 (11–18), *p* = 0.264) (Supplemental Figure S3, Supplementary Table S1).

### Mortality stratification by I-FABP and identification as a predictor for 28d-mortality

**Fig. 4:**
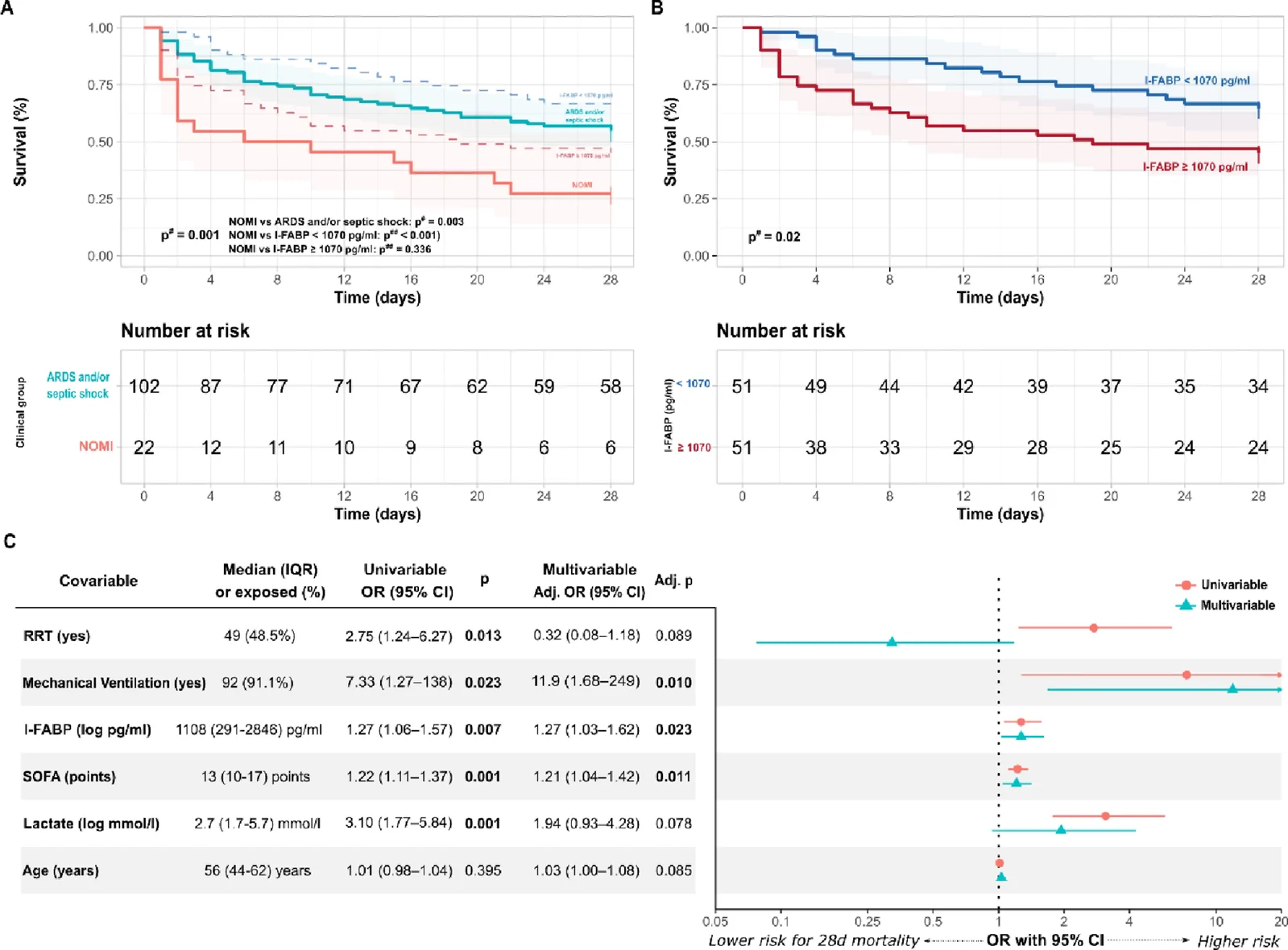
28-day survival stratified by I-FABP. Survival is shown as Kaplan-Meier graphs for (**A**) patients divided by clinical groups (patients with NOMI and patients with ARDS and/or septic shock). Dotted lines indicate divided ARDS and/or septic shock group into low- and high- I-FABP (threshold 1070 pg/ml). (**B**) Patients with ARDS and/or septic shock stratified by low or high I-FABP concentrations are shown as Kaplan-Meier graphs. (**C**) Univariate and multivariate logistic regression were performed for patients with septic shock and/or ARDS to identify predictors for 28-day mortality and odds ratios (OR) with corresponding 95% confidence intervals (CI) are shown. p-values < 0.05 were considered significant. ^#^ = log-rank test, ^##^ = post-hoc log-rank test

In patients with septic shock and/or ARDS, 44 out of 102 patients (43%) died on the ICU within 28 days. In the NOMI cohort 28-day-mortality was overall significantly higher (i.e. 73%, log rank test: *p* = 0.003) (Fig. 4A). While NOMI patients had a significant higher mortality compared to ARDS and/or septic shock patients with low I-FABP (*p* < 0.001), mortality was not different between high I-FABP and NOMI patients in a post-hoc log-rank test (Fig. 4A). Of note, if stratified for the I-FABP threshold in patients with ARDS and/or septic shock, high I-FABP concentrations were associated with an increased mortality rate (52.9%) compared to lower I-FABP concentrations (33.3%, log rank test: *p* = 0.02) (Fig. 4B).

To further analyze the impact of I-FABP levels on mortality in patients with ARDS and/or septic shock, we performed a univariate logistic regression for 28-day-mortality in this cohort to address possible bias due to informative censoring. Adjustment variables were selected a priori based on their established clinical relevance as determinants of mortality in critically ill patients. SOFA score, lactate concentrations, need of RRT or mechanical ventilation showed an increased mortality (e.g. I-FABP: Odds ratio (OR): 1.27 (95% CI 1.06–1.58), *p* = 0.007) (Table 2, Supplementary Table S2). In a multivariate logistic regression, higher I-FABP remained an independent predictor for 28-day-mortality (adjusted OR for I-FABP: 1.27 (1.03–1.62), *p* = 0.023) (Table 2; Fig. 4C, Supplementary Table S2). SOFA score also remained an independent predictor for 28d-mortality, whereas lactate concentrations were not independently associated with higher 28d-mortality in this multivariate logistic regression (Fig. 4C, Supplementary Table S2). The number of mortality events per predictor parameter (EPV) was 7.3. Concerning model calibration and discrimination the apparent AUC was 0.811 and the Brier score was 0.174 (Supplementary Table S3). Of note, after additional adjustment for TPE, higher I-FABP remained independently associated with 28-day mortality (adjusted OR for I-FABP: 1.25 (1.01–1.61), *p* = 0.042). TPE was also associated with higher mortality (Supplementary Table S2).

**Table 2:** Associations between I-FABP and 28-day mortality

Model	OR	95% CI	*p*
Unadjusted	1.735	1.153–2.846	**0.007**
Adjusted for age, lactate, SOFA score, mechanical ventilation and RRT	1.593	1.022–2.703	**0.039**
Adjusted for age, lactate, SOFA score, mechanical ventilation, RRT and TPE	1.254	1.008–1.613	**0.042**

Logistic regressions were performed to analyze the associations between I-FABP and 28-day mortality unadjusted and adjusted for Age, SOFA score, RRT, mechanical ventilation and lactate concentration in patients with septic shock and/or ARDS. Additionally, adjustment for TPE was performed. Odds ratios (OR) are shown with corresponding 95%-confidence intervals. P-values < 0.05 were seen as significant and displayed in bold.

I-FABP – intestinal Fatty-acid binding protein, RRT – renal replacement therapy, TPE – therapeutic plasma exchange

## Discussion

In this study, we demonstrated that circulating I-FABP concentrations at ICU admission were markedly elevated in patients with ARDS and/or septic shock, similar to those observed in NOMI patients, compared with healthy individuals. Using ROC analysis from the REPERFUSE cohort of patients with angiography-based diagnosis of NOMI as endpoint, a cutoff value of 1070 pg/ml best discriminated significant ischemic intestinal injury. In critical-ill patients (with ARDS and/or septic shock), I-FABP concentrations above this threshold were associated with higher lactate levels, greater norepinephrine requirement, and an increased 28-day mortality. These findings indicate that I-FABP may reflect intestinal injury, supporting its potential role as a prognostic biomarker in critically ill patients.

One of our key goals was to identify a clinically relevant I-FABP threshold for likely ischemic intestinal injury. Using a reference cohort of patients with angiography-confirmed NOMI from the REPERFUSE study and healthy individuals, an optimal Youden’s index was identified at 1070 pg/ml with a specificity of 100% and sensitivity of 68%. This approach provides a pragmatic threshold for “low” versus “high” I-FABP in our cohort of critical ill patients with ARDS and/or septic shock.

In prior literature, I-FABP cutoffs have varied widely depending on the context and etiology of intestinal ischemia. For example, in patients with strangulated small-bowel obstruction, *Kittaka et al.*. reported a cutoff of 6500 pg/ml for discriminating strangulation and simple small-bowel obstruction [35]. In two meta-analyses, pooled diagnostic accuracy for I-FABP for acute mesenteric ischemia (AMI) were analyzed. Treskes et al. distinguished between different ELISA kits (Uden and Osaka kit). For the Uden kit, which was used for our analyses as well, a cutoff range between 0.09 and 0.815 ng/ml was found with a pooled sensitivity of 79.0% and a pooled specificity of 91.3% for I-FABP’s diagnostic accuracy for acute intestinal ischemia. On the contrary, overall higher cutoffs (3.1–100 ng/ml) were found in the Osaka ELISA kit, however, resulting in lower pooled sensitivity (75%) and pooled specificity (79.2%) [29]. Sun et al. found a pooled sensitivity of 80% and a pooled specificity of 85% for I-FABP’s diagnostic accuracy, with cutoffs between 0.1 and 101.1 ng/ml. However, it was not distinguished between different ELISA kits. As the pooled sensitivity and specificity are between the individual ELISA kit results from Treskes et al., presumably results from both ELISA kits were included, although details on used kits are not stated [17]. Our results are especially comparable to the pooled results reported by the Uden kit with a low cutoff. One more recent cohort looking at patients after cardiac surgery found an ideal cutoff of 1421 pg/ml for detecting early mesenteric ischemia (AUC 0.973), comparable to our results [36]. However, work concerning a cutoff for I-FABP in septic and ARDS patients is overall scarce [25, 28, 37], especially in ARDS patients [8]. Our threshold of 1070 pg/ml is lower than some previous cutoffs in overt surgical ischemia but may be appropriate to identify early intestinal injury rather than bowel necrosis. External validation in an independent, preferably age-matched ICU population is required to confirm the generalizability of the proposed cutoff.

Our findings demonstrate that plasma I-FABP concentrations are elevated in patients with septic shock and/or ARDS and can reach levels comparable with patients with diagnosed NOMI, even in the absence of overt mesenteric ischemia. Elevated I-FABP concentrations in patients with septic shock and sepsis compared to healthy controls were previously described [38]. In other critical conditions (e.g. COVID-19 or meningococcal septic children) elevated I-FABP levels have also been reported [39, 40]. These observations indicate that elevated circulating I-FABP is common in critical illness, even in the absence of clinically apparent mesenteric ischemia, and raise the hypothesis of subclinical intestinal epithelial injury [25]. I-FABP may serve as an early indicator of intestinal injury in this “subclinical” phase and may trigger earlier vigilance for gastrointestinal complications or even therapeutic measures. A defined threshold indicating significant intestinal injury to identify high risk patients is therefore highly desirable.

In our cohort, patients with I-FABP above the identified threshold had significantly higher lactate concentrations and needed higher norepinephrine doses, suggesting that elevated I-FABP may be associated with systemic hypoperfusion and vasopressor-dependent shock in critically ill patients. Importantly, I-FABP concentrations were not elevated in the subgroup of ARDS patients without shock compared with healthy individuals. This observation is consistent with the hypothesis that circulatory failure and splanchnic hypoperfusion may contribute more strongly to I-FABP elevation than lung injury per se. Accordingly, Tyszko et al. found that I-FABP levels were higher in patients with septic shock compared to sepsis without shock [16]. Interestingly, in previous work endotoxin levels in septic shock did not correlate with I-FABP levels, suggesting that the gut injury might be more perfusion- than endotoxin-driven [41]. Supporting that splanchnic hypoperfusion and shock are central for enterocyte injury, patients with various etiologies of shock, e.g. cardiogenic shock and acute heart failure [42] as well as patients with heat shock [43] also showed elevated I-FABP levels. These findings align with prior observations from our group that intestinal perfusion is particularly vulnerable to catecholamine-induced vasoconstriction in shock [14]. Therefore, I-FABP could help identify patients with early, clinically undetected gut injury and thus potentially guide early monitoring, further diagnostic- (e.g. imaging) and possibly even therapeutic interventions (e.g. intra-arterial vasodilatory therapy) aiming towards early optimization of splanchnic perfusion in critically ill patients [14, 44]. Importantly, our and other studies suggest that gut injury in critical illness is not necessarily a late complication but may occur as early as at ICU admission in shock, where it already is strongly associated with later ICU mortality [45, 46].

One perhaps surprising finding is the absence of a significant change in I-FABP levels over approximately three days in a subset of our patients. This may indicate that intestinal injury occurs early, but I-FABP kinetics may be subject to changes in kidney function (accumulation in dysfunction), high-cutoff RRT (higher elimination rates) and its short half-time of <1 h [47, 48]. Additionally, patients who received TPE on the day of inclusion did not have lower I-FABP levels during follow-up, which might suggest only temporarily reduction directly after TPE and continuous release of I-FABP potentially due to prolonged shock and higher shock severity. Other cohorts have shown I-FABP elevations over days 1 and 3 in septic shock [16]. Thus, while I-FABP may detect early injury, its dynamic behavior in ICU patients clearly needs further investigations ideally combined with other biomarkers (e.g., citrulline, SM22) [16].

In our cohort, 28-day mortality was 43%. Higher I-FABP concentrations were independently associated with 28-day mortality after adjustment for variables that were selected a priori based on their established clinical relevance as determinants of mortality in critically ill patients. As residual confounding cannot be excluded, this association should be interpreted cautiously. The model demonstrated good apparent discrimination, with a Brier score of 0.174 and a Nagelkerke R² of 0.385. However, given the limited number of mortality events relative to model complexity, potential overfitting cannot be excluded. Our results are consistent with a previous prospective observational study by Sekino et al. in which I-FABP was associated with incidence of NOMI, as well as 28-day mortality [45].

Notably, SOFA score remained an independent predictor of 28-day mortality in the multivariable model, indicating that the association between elevated I-FABP concentrations and mortality was not merely a reflection of overall illness severity as captured by established organ dysfunction scores. This interpretation is further supported by the absence of significant differences in SOFA scores between patients with low and high I-FABP concentrations. Moreover, generalized additive model analyses demonstrated only a non-linear association between I-FABP concentrations and established markers of shock severity (lactate concentrations and norepinephrine dose). While both parameters increased with rising I-FABP concentrations in the lower and intermediate range, these relationships plateaued at higher I-FABP levels, suggesting that I-FABP is not simply a surrogate marker of circulatory failure or tissue hypoperfusion. Consistent with this notion, lactate concentrations were not independently associated with 28-day mortality in the multivariable model. As TPE was substantially more frequent among patients with higher I-FABP and thereby potentially influencing the association of I-FABP and 28-day mortality, an additional analysis adjusting for TPE was performed, where higher I-FABP remained independently associated with mortality. Of note, the association between TPE and mortality should not be interpreted causally, as TPE was preferentially used in patients with more severe disease, resulting in confounding by indication. Taken together, these findings suggest that I-FABP may provide prognostic information complementary to conventional measures of organ dysfunction and shock severity. Whether this association reflects intestinal epithelial injury, barrier dysfunction, or other pathophysiological processes of critical illness remains uncertain and requires prospective mechanistic investigation.

This study has several limitations. First, it was a post hoc analysis combining data from different prospective cohorts, introducing potential selection bias and heterogeneity, especially as some patients received TPE as a possible confounder. The sample size of the NOMI reference group was limited, which may have affected the precision of the identified cutoff value. Additionally, in the NOMI cohort gut injury was confirmed by systematic imaging assessment, whereas in the ARDS and/or septic shock cohort extend of gut injury was not quantified. Accordingly, elevated circulating I-FABP cannot be considered direct evidence of intestinal barrier dysfunction in this cohort, and the underlying biological mechanisms of its association with mortality remain uncertain. The comparison with healthy controls may have overestimated the diagnostic performance of I-FABP, while elevated concentrations in critically ill patients without confirmed NOMI indicate limited specificity for the diagnosis of NOMI. Additionally, healthy controls were significantly younger compared to the other cohorts and an influence of age on baseline I-FABP variability cannot be excluded and may have affected both the comparison with healthy controls and the ROC-derived cutoff. Therefore, the proposed threshold requires external validation, ideally in age-matched populations. The lack of dynamic I-FABP monitoring beyond day 3 precludes insights into long-term kinetics or mucosal recovery. Lastly, although I-FABP correlated with mortality and shock severity, future prospective studies are needed to validate I-FABP as a diagnostic and therapeutic decision tool in critically ill patients.

## Conclusions

In conclusion, circulating I-FABP levels are elevated in patients with septic shock and/or ARDS, but not in ARDS patients without concomitant septic shock, supporting a potential association between I-FABP elevation, shock, and impaired intestinal perfusion. A cutoff of 1070 pg/ml might identify patients already at ICU admission who are at risk for intestinal ischemia. I-FABP may provide prognostic information beyond that captured by conventional measures of organ dysfunction or shock severity. Prospective studies are required to determine whether I-FABP specifically reflects intestinal epithelial or barrier dysfunction and whether its incorporation into multimodal assessment strategies facilitates earlier detection of intestinal injury and identification of candidates for targeted intervention.

## Supplementary Information

Below is the link to the electronic supplementary material.

Supplementary Material 1

Supplementary Material 3

Supplementary Material 4

Supplementary Material 5

Supplementary Material 5

Supplementary Material 6

Supplementary Material 7

## Data Availability

The data underlying this article will be shared on reasonable request to the corresponding author.

## Declarations

### AI statement

Artificial intelligence tools were used to assist with language refinement and grammar checking in the preparation of this manuscript. The authors reviewed and edited all content and take full responsibility for the final version of the text.

## Abbreviations

ARDS: Acute respiratory distress syndrome
AUC: Area under the curve
BMI: Body mass index
CI: Confidence interval
CAD: Coronary artery disease
CKD: Chronic kidney disease
COPD: Chronic obstructive pulmonary disease
CRP: C–reactive protein
EPV: Events per variable
OR: Odds Ratio
I-FABP: Intestinal fatty acid binding protein
IQR: Interquartile range
NOMI: Non–occlusive mesenterial ischemia
PCT: Procalcitonin
ROC: Receiver operating characteristic
RRT: Renal replacement therapy
SOFA: Sequential organ failure assessment
WBC: White blood cell
