# Supplementary material for: Intestinal fatty acid-binding protein (I-FABP) at ICU admission as early biomarker of intestinal injury and predictor of mortality in septic shock
Source: Intensive Care Med Exp. 2026 Aug 26;14:112. doi: 10.1186/s40635-026-00963-9 (PMC13518634; doi:10.1186/s40635-026-00963-9)
Supplement: Supplementary file 1 [file 40635_2026_963_MOESM1_ESM.docx]

**Supplemental Material**

**Supplemental Material 1**

Collected variables included demographic characteristics (age, sex, body mass index), comorbidities (obesity, hypertension, diabetes mellitus, chronic obstructive pulmonary disease, heart failure, coronary artery disease, and chronic kidney disease), site of infection, organ support measures (renal replacement therapy and mechanical ventilation), hemodynamic variables (norepinephrine dose), laboratory parameters (lactate, C-reactive protein, procalcitonin, and white blood cell count), and respiratory variables including the oxygenation index (PaO₂/FiO₂ ratio).

All cohorts were established and maintained by the same study team and data were extracted from the PDMS using identical variable definitions and units across cohorts. For example, norepinephrine dose was recorded uniformly in µg/kg/min and laboratory parameters were reported using the same institutional standards. Because I-FABP and lactate concentrations exhibited a right-skewed distribution, values were logarithmically transformed prior to analysis and entered as a continuous variable. Prior to merging the datasets, variable names, coding schemes, and units were verified for consistency. As no relevant differences in data structure or measurement units were identified, the cohort datasets were merged into a single harmonized database for the present analysis. Patients were followed throughout their hospital stay, and mortality was recorded until discharge or death.
