# Supplementary material for: Intestinal fatty acid-binding protein (I-FABP) at ICU admission as early biomarker of intestinal injury and predictor of mortality in septic shock
Source: Intensive Care Med Exp. 2026 Aug 26;14:112. doi: 10.1186/s40635-026-00963-9 (PMC13518634; doi:10.1186/s40635-026-00963-9)
Supplement: Supplementary file 2 [file 40635_2026_963_MOESM5_ESM.docx]

| **Suppl. Table 1**: Demographic and clinical characteristics depending on I-FABP | | | | |
| --- | --- | --- | --- | --- |
| **Category (median (IQR) or N (%))** | **Low I-FABP** n=51 | **High I-FABP** n=51 |  | **p** |
| Age (years) | 54 (43-62) | 56 (47-63) |  | 0.663^#^ |
| Sex (female) | 17 (33.3) | 15 (29.4) |  | 0.670^‡^ |
| BMI (kg/m^2^) | 26.0 (23.1-29.3) | 24.9 (22.6-30.7) |  | 0.649^#^ |
| Comorbidities |  |  |  |  |
| Adipositas | 15 (33.3) | 13 (33.3) |  | 1.000^‡^ |
| Hypertension | 16 (34.8) | 17 (43.6) |  | 0.691^‡^ |
| Diabetes | 8 (17.4) | 9 (23.1) |  | 0.796^‡^ |
| COPD | 7 (15.2) | 3 (7.7) |  | 0.561^‡^ |
| Heart insufficiency | 7 (15.6) | 7 (17.9) |  | 0.951^‡^ |
| CAD | 6 (13.0) | 2 (5.1) |  | 0.460^‡^ |
| CKD | 13 (29.5) | 7 (17.9) |  | 0.468^‡^ |
| Site of infection |  |  |  | 0.494^‡^ |
| Pulmonary | 34 (75.6) | 30 (63.9) |  | 0.240^‡^ |
| Abdominal | 6 (13.3) | 10 (21.3) |  | 0.420^‡^ |
| Soft tissue | 2 (4.4) | 4 (8.5) |  | 0.680^‡^ |
| Endocarditis | 1 (2.2) | 1 (2.1) |  | 1.000^‡^ |
| SOFA score (points) | 13 (10-17) | 15 (11-18) |  | 0.264^#^ |
| Norepinephrine dose (µg/kg/min) | 0.469 (0.272-0.786) | 0.675 (0.459-0.932) |  | **0.017**^#^ |
| Lactate concentration (mmol/l) | 2.4 (1.4-3.5) | 3.9 (2.2-8.9) |  | **< 0.001**^#^ |
| RRT | 19 (37.3) | 30 (60.0) |  | **0.022**^‡^ |
| TPE | 18 (35.3) | 31 (60.8) |  | **0.010** |
| Mechanical ventilation | 49 (96.1) | 43 (86.0) |  | 0.075^‡^ |
| Oxygenation index (PaO_2_/FiO_2_) (mmHg) | 130 (94-202) | 118 (75-167) |  | 0.223^#^ |
| CRP (mg/l) | 221 (150-305) | 213 (130-330) |  | 0.997^#^ |
| PCT (µg/l) | 19.9 (2.3-41.8) | 15.2 (3.4-55.5) |  | 0.579^#^ |
| WBC (10^3^/µl) | 13.1 (6.9-19.3) | 9.9 (6.0-17.6) |  | 0.316^#^ |

Abbreviations: BMI - Body mass index, CAD – coronary artery disease, CKD – chronic kidney disease, COPD – chronic obstructive pulmonary disease, CRP - C-reactive protein, IQR - Interquartile range, PCT - Procalcitonin, RRT - Renal replacement therapy, SOFA - Sequential Organ Failure Assessment, TPE – Therapeutic plasma exchange, WBC - White blood cell. #=Mann-Whitney-U test; ‡=Chi-Squared test.
