# Supplementary material for: Intestinal fatty acid-binding protein (I-FABP) at ICU admission as early biomarker of intestinal injury and predictor of mortality in septic shock
Source: Intensive Care Med Exp. 2026 Aug 26;14:112. doi: 10.1186/s40635-026-00963-9 (PMC13518634; doi:10.1186/s40635-026-00963-9)
Supplement: Supplementary file 3 [file 40635_2026_963_MOESM6_ESM.docx]

| **Supplementary Table S2:** Predictors of 28-day mortality | | | | | | |  | |  |  | |  | | |  | | |  | | |  |  | |  |
| --- | --- | --- | --- | --- | --- | --- | --- | --- | --- | --- | --- | --- | --- | --- | --- | --- | --- | --- | --- | --- | --- | --- | --- | --- |
|  |  |  | | **28d-mortality** | | | | | | | | | | |  | | | | | | | | **28d-mortality** | |
|  |  |  |  |  |  |  |  |  |  |  |  |  |  |  |  |  |  |  |  |  |  |  |  |  |
| **characteristic** |  |  | **univariate** | |  |  | |  | | | **multivariate** | |  |  | |  | **multivariate** | |  |  |  |  |  |  |
|  |  |  |  | |  |  | |  | | |  | |  |  | |  |  | |  |  |  |  |  |  |
|  |  | **OR** | **95% CI** | | **p** |  | | **OR** | | | **95% CI** | | **p** |  | | **OR** | **95% CI** | | **p** |  |  |  |  |  |
|  |  |  |  | |  |  | |  | | |  | |  |  | |  |  | |  |  |  |  |  |  |
| Age (years) |  | 1.013 | 0.984 – 1.044 | | 0.395 |  | | 1.033 | | | 0.996 – 1.077 | | 0.085 |  | | 1.035 | 0.996 – 1.080 | | 0.083 |  |  |  |  |  |
| Lactate (log mmol/l) |  | 3.104 | 1.774 – 5.839 | | **< 0.001** |  | | 1.937 | | | 0.929 – 4.276 | | 0.078 |  | | 1.499 | 0.676 – 3.457 | | 0.322 |  |  |  |  |  |
| SOFA score (points) |  | 1.224 | 1.110 – 1.367 | | **< 0.001** |  | | 1.209 | | | 1.043 – 1.424 | | **0.011** |  | | 1.184 | 1.006 – 1.419 | | **0.042** |  |  |  |  |  |
| I-FABP (log pg/ml) |  | 1.270 | 1.064 – 1.575 | | **0.007** |  | | 1.267 | | | 1.031 – 1.617 | | **0.023** |  | | 1.254 | 1.008 – 1.613 | | **0.042** |  |  |  |  |  |
| Mechanical Ventilation (yes) |  | 7.333 | 1.272 – 138.84 | | **0.023** |  | | 11.93 | | | 1.684 – 249.36 | | **0.010** |  | | 15.157 | 2.037 – 321.99 | | **0.006** |  |  |  |  |  |
| RRT (yes) |  | 2.745 | 1.235 – 6.273 | | **0.013** |  | | 0.324 | | | 0.077 – 1.182 | | 0.089 |  | | 0.261 | 0.053 – 1.055 | | 0.060 |  |  |  |  |  |
| TPE (yes) |  | 7.744 | 3.285 – 19.438 | | **0.001** |  | | - | | | - | | - |  | | 5.105 | 1.780 – 15.734 | | **0.002** |  |  |  |  |  |

ABBREVIATIONS:

I-FABP – intestinal Fatty-acid binding protein, RRT – renal replacement therapy, TPE – therapeutic plasma exchange
