# Supplementary material for: Intestinal fatty acid-binding protein (I-FABP) at ICU admission as early biomarker of intestinal injury and predictor of mortality in septic shock
Source: Intensive Care Med Exp. 2026 Aug 26;14:112. doi: 10.1186/s40635-026-00963-9 (PMC13518634; doi:10.1186/s40635-026-00963-9)
Supplement: Supplementary file 4 [file 40635_2026_963_MOESM7_ESM.docx]

| **Supplementary Table S3**: Model calibration and discrimination for logistic regression model for 28-day mortality | | |
| --- | --- | --- |
| **Domain** | **Variable** | **Estimate** |
| Model complexity | Predictor parameters | 6 |
| Model stability | EPV | 7.3 |
| Model discrimination | AUC with 95% CI | 0.811 (0.726-0.897),  p < 0.001 |
| Overall predictive performance | Brier score | 0.174 |
|  | Null model Brier score | 0.245 |
| Model fit | Nagelkerke R^2^ | 0.385 |
|  | Cox-Snell R^2^ | 0.289 |

Abbreviations: AUC - area under the receiver operating characteristic curve; CI - confidence interval, EPV – Events per variable.
